# 2357. Descriptive Analyses of mRNA-1273 (Moderna COVID-19 vaccine) Spontaneous Reports by Geographic Regions

**DOI:** 10.1093/ofid/ofad500.1978

**Published:** 2023-11-27

**Authors:** Magalie Emile-Backer, Vaishali Khamamkar, Priyadarshani Dharia, Samantha St Laurent, Cindy Ke Zhou, Daina Esposito, David Martin, Walter Straus

**Affiliations:** Moderna, Inc., Cambridge, Massachusetts; Moderna, Inc., Cambridge, Massachusetts; Moderna, Inc., Cambridge, Massachusetts; Moderna, Inc., Cambridge, Massachusetts; Moderna, Inc., Cambridge, Massachusetts; Moderna, Inc., Cambridge, Massachusetts; Moderna, Inc., Cambridge, Massachusetts; Moderna, Inc., Cambridge, Massachusetts

## Abstract

**Background:**

Given global distribution of the mRNA-1273 vaccine in the pandemic, an understanding of regional differences in adverse event (AE) reporting is needed.

**Methods:**

The Moderna global safety database (GSDB) contains mRNA-1273 associated AE reports directly submitted by health care providers and consumers, as well as from company supported trials and observational studies, reports received from regulators (80%), and identified in the literature. We evaluated AE reports by geographic region using a cross-sectional descriptive analysis.

**Results:**

As of 17 Feb 2023, 1,654,338,053 doses of mRNA-1273 had been distributed. The greatest proportion of doses were in North America (40.2%), Europe (28.9%), and Asia (21.9%). Africa, Middle East, Latin America and Oceania represented < 10% of doses distributed. A total of 2,622,777 events (0.3%) were reported in 686,515 cases. Most cases were reported from Europe (52%) and North America (39.0%). The lowest proportion of reported cases were from Africa and the Middle East (< 0.01% and 0.03%, respectively) (Figure 1). In these regions, a higher proportion of cases were reported for children (74.0% and 44.0%, respectively) compared to the US (14.3%) and Europe (6.2%). The proportion of cases meeting the standard pharmacovigilance definition of *‘serious’* also varied (Figure 2). Across all regions, 49.0% of reported adverse events were indicative of reactogenicity, defined as commonly “expected” vaccine-related reactions (e.g., headache, pyrexia, fatigue). This proportion was highest in Europe (57.0%) and Latin America (59.0%) and lowest in Africa (6.1%). Across all regions, 59.0% of cases did not document medical history, and this was most common ( > 85%) for reports from Africa, Latin America, Asia and Oceania.

**Figure:**
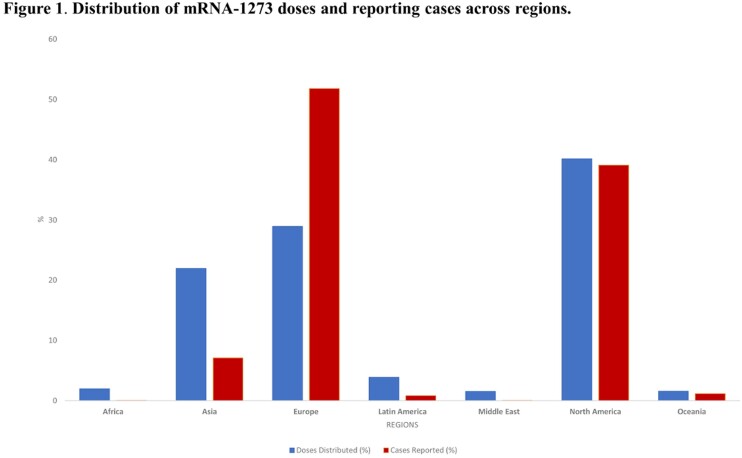

**Conclusion:**

Regional differences in frequency and data quality for spontaneous reporting of adverse events was evident. Differences in reporting may be attributable to variability in local reporting infrastructure. Where appropriate, active surveillance studies may supplement spontaneous data to allow for more robust vaccine safety monitoring across regions.

**Figure:**
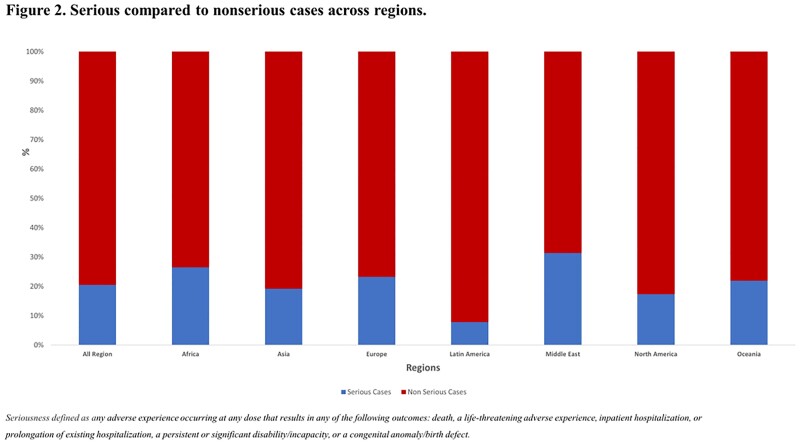

**Disclosures:**

**Magalie Emile-Backer, PharmD, CCRP**, Moderna, Inc.: Salary|Moderna, Inc.: Stocks/Bonds **Vaishali Khamamkar, MS**, Moderna, Inc.: Salary|Moderna, Inc.: Stocks/Bonds **Priyadarshani Dharia, PhD, MD, MPH**, Moderna, Inc.: Salary|Moderna, Inc.: Stocks/Bonds **Samantha St Laurent, MPH**, Moderna, Inc.: Salary|Moderna, Inc.: Stocks/Bonds **Cindy Ke Zhou, PhD, MBBS**, Moderna, Inc.: Salary|Moderna, Inc.: Stocks/Bonds **Daina Esposito, PhD, MPH**, Moderna, Inc.: Salary|Moderna, Inc.: Stocks/Bonds **David Martin, MD, MPH**, Moderna, Inc.: Salary|Moderna, Inc.: Stocks/Bonds **Walter Straus, MD, MPH**, Moderna, Inc.: Salary|Moderna, Inc.: Stocks/Bonds

